# Improvement in Touch Sensation after Stroke is Associated with Resting Functional Connectivity Changes

**DOI:** 10.3389/fneur.2015.00165

**Published:** 2015-07-31

**Authors:** Louise C. Bannister, Sheila G. Crewther, Maria Gavrilescu, Leeanne M. Carey

**Affiliations:** ^1^Neurorehabilitation and Recovery, Stroke Division, Florey Institute of Neuroscience and Mental Health, Melbourne, VIC, Australia; ^2^School of Psychology and Public Health, College of Science, Health and Engineering, La Trobe University, Melbourne, VIC, Australia; ^3^Occupational Therapy, School of Allied Health, College of Science, Health and Engineering, La Trobe University, Melbourne, VIC, Australia; ^4^Defence Science and Technology Organisation, Melbourne, VIC, Australia; ^5^Florey Department of Neuroscience and Mental Health, The University of Melbourne, Melbourne, VIC, Australia

**Keywords:** stroke recovery, somatosensory disorders, neuronal plasticity, magnetic resonance imaging, tactile, intrinsic functional connectivity

## Abstract

**Background:**

Distributed brain networks are known to be involved in facilitating behavioral improvement after stroke, yet few, if any, studies have investigated the relationship between improved touch sensation after stroke and changes in functional brain connectivity.

**Objective:**

We aimed to identify how recovery of somatosensory function in the first 6 months after stroke was associated with functional network changes as measured using resting-state connectivity analysis of functional magnetic resonance imaging (fMRI) data.

**Methods:**

Ten stroke survivors underwent clinical testing and resting-state fMRI scans at 1 and 6 months post-stroke. Ten age-matched healthy participants were included as controls.

**Results:**

Patients demonstrated a wide range of severity of touch impairment 1 month post-stroke, followed by variable improvement over time. In the stroke group, significantly stronger interhemispheric functional correlations between regions of the somatosensory system, and with visual and frontal areas, were found at 6 months than at 1 month post-stroke. Clinical improvement in touch discrimination was associated with stronger correlations at 6 months between contralesional secondary somatosensory cortex (SII) and inferior parietal cortex and middle temporal gyrus, and between contralesional thalamus and cerebellum.

**Conclusion:**

The strength of connectivity between somatosensory regions and distributed brain networks, including vision and attention networks, may change over time in stroke survivors with impaired touch discrimination. Connectivity changes from contralesional SII and contralesional thalamus are associated with improved touch sensation at 6 months post-stroke. These functional connectivity changes could represent future targets for therapy.

## Introduction

Somatosensory impairment is common after stroke, occurring in 50–80% of stroke survivors (1, 2). However, investigations of the neural correlates of clinical somatosensory improvement after stroke are scarce (3). In particular, knowledge of how brain networks are interrupted is limited, but is critical to better understand the nature of the clinical deficit and post-stroke recovery (4).

Stroke impacts not only the focal lesion site but also on remote brain regions (5, 6). Lesions have important remote effects on the function of connected neural networks that are structurally intact, i.e., physiological changes in distant but functionally related brain areas (4, 7, 8). These remote effects contribute significantly to the observed behavioral deficits and recovery potential (4, 8). Further, changes in brain networks (across both hemispheres and function-specific networks) have been shown to be important in recovery of motor and attention functions (4, 6). A significant challenge is to identify the brain networks and processes that mediate functional improvement so that rehabilitation strategies can be aimed at the appropriate targets (9).

Only a few studies have investigated changes in the brain over time in association with somatosensory recovery (3, 10–13). These studies have primarily involved identification of brain regions associated with task-related brain activation. A few studies have reported that somatosensory recovery is associated with patterns of activation in primary somatosensory (SI) cortex that resembles those seen in healthy controls. For example, return of ipsilesional SI activation has been shown to be associated with improved somatosensory perception (10–12). Staines et al. (12) found that enhanced primary somatosensory cortex activation using functional MRI in the stroke-affected hemisphere occurred in conjunction with improved touch detection in four patients with thalamocortical strokes. Likewise, Wikström et al. (10) reported that increased amplitude of early somatosensory evoked fields in the ipsilesional SI in response to median nerve simulation was associated with recovery of two-point discrimination (the ability to discern that two nearby objects touching the skin are truly two distinct points, not one) in stroke patients.

While relative “normalization” of brain activity in primary and secondary (SII) somatosensory regions in both hemispheres seems to underlie good clinical recovery, patients with more severe impairments have been shown to recruit attention and multisensory brain regions to a greater degree than that seen in healthy controls, in order to accomplish successful task performance (3, 11, 14–17). In an early positron emission tomography (PET) study of five patients after subcortical stroke, Weder et al. (14) reported activation across bilateral sensorimotor cortex and distributed regions, such as premotor cortex and cerebellum, with worse performance on a tactile shape discrimination task found to correlate with bilateral sensorimotor cortex activation. Tecchio et al. (16) used magnetoencephalography (MEG) to study 18 patients at the acute (5 days) and post-acute (6 months) stages after stroke. They reported that excessive interhemispheric asymmetry correlated with a greater degree of clinical improvement over time in those patients who showed partial recovery. Taskin et al. (15) reported reduced activation of ipsilesional SI with preserved responsiveness of SII in six patients who had suffered thalamic strokes. More recently, in 19 patients, a study into the relationship between touch impairment and interruption to cortical and subcortical somatosensory areas revealed that the neural correlates of touch impairment in patients with interruption to subcortical somatosensory areas (e.g., thalamus), involved a distributed network of ipsilesional SI and SII, contralesional thalamus, and attention-related frontal and occipital regions (3).

Use of task-based brain activation paradigms can be challenging for stroke patients who may have difficulty performing a given task, and inability to perform the task may impact on the validity of the results (18). Resting-state functional connectivity analysis of functional magnetic resonance imaging (fMRI) data has more recently been employed as a way of assessing activity in the brain over time and across different networks of the brain (19, 20). Resting-state functional connectivity reveals intrinsic, spontaneous networks that elucidate the functional architecture of the human brain at rest (task-independent). Functional connectivity is defined as the statistical association (or temporal correlation) among two or more anatomically distinct regions (21). Data are analyzed for coherence across the whole brain and/or in relation to particular regions of interest (ROIs). Evidence suggests that this measure is indicative of behaviorally relevant brain networks without requiring task performance (22). Consistent resting-state networks, with sharp transitions in correlation patterns, are reliably detected in individual and group data (23, 24).

In stroke patients, use of this technique has revealed disruption of functional connectivity of brain networks, even within structurally intact brain regions (6, 25, 26). Changes in functional connectivity have been described in motor recovery under resting-state and task-related conditions (27). Further, changes in functional connectivity over time have been found to occur in conjunction with behavioral change, both in healthy individuals (22) and in stroke patients (7, 25). For example, He and colleagues (25) reported that in patients with spatial neglect, dorsal attention network connectivity was disrupted early after stroke, but appeared to have improved to similar levels as controls by 9 months post-stroke, in conjunction with behavioral improvement. This supports the interpretation that different networks or areas of the brain may dynamically change and assume different roles to allow behavior to occur.

The aim of the current study was to identify longitudinal changes in functional connections of the somatosensory network in stroke patients with somatosensory impairment, and to establish if and how these correlations are associated with improvement in touch discrimination.

The importance of interhemispheric functional connectivity in behavioral performance and recovery has been highlighted from studies using resting-state fMRI (rsfMRI) with animal and human stroke populations (7, 25, 28). The most consistent finding is of changes in interhemispheric functional connectivity between homotopic areas, such as ipsilesional and contralesional primary motor cortex (7). Longitudinal changes have also been reported. Decreased interhemispheric functional connectivity of the ipsilesional sensorimotor cortex has been reported early after stroke, with return to more normal levels during the recovery process (7, 29, 30). These findings are not surprising given that interhemispheric connections are implicated in sensory (31) and cognitive processing (32) and in models of motor and somatosensory recovery (33–37). Thus, changes in interhemispheric functional connectivity in stroke patients and associations between these changes and behavioral improvement are expected. We hypothesized that over time, stroke patients would exhibit return to a more “typical” pattern of interhemispheric functional connectivity between homologous cortical somatosensory regions, and that stronger interhemispheric resting-state functional correlations between homologous SI and SII regions at 6 months than at 1 month post-stroke would be associated with clinical improvement.

Increased connectivity with distributed networks has also been reported in recovery after stroke. First, the visual system drives human attention and planning (38, 39), and a rich history of evidence for cross-modal plasticity between the visual and somatosensory systems exists (40). Recruitment of visual areas has been reported in previous studies of motor recovery after stroke (30, 41) as well as in patients with somatosensory impairment after stroke (3). Second, greater recruitment of attention systems is known to be necessary (42) to compensate for the impairment of function-specific brain areas due to aging or injury (43, 44). In stroke patients, increased attention has been shown to be required to accomplish previously simple tasks, such as walking, and attention skills have been shown to predict outcome after stroke (42, 45). Increased activation of frontoparietal attention areas, such as inferior parietal cortex (IPC), has been reported to occur in recovering stroke patients with motor problems (46–48). Thus, greater functional connections with frontoparietal attention networks could be expected in stroke patients with somatosensory impairment. As such, we predicted that stronger thalamocortical and cortico-cortical functional correlations with frontoparietal visual attention networks at 6 months post-stroke would be associated with clinical improvement.

## Materials and Methods

### Participants

Ten stroke patients with impaired touch discrimination of the upper limb were assessed at 1 and 6 months post-stroke. Inclusion criteria were as follows: first episode infarct, medical stability, ability to give informed consent and comprehend simple instructions, and right-hand dominance. Exclusion criteria included the following: brain-stem infarct or hemorrhagic stroke, previous neurological dysfunction, medical history impairing hand function or precluding MRI, or evidence of neglect based on standard neuropsychological tests. We also studied 10 age-matched, right-hand dominant healthy controls (4 male, mean age 60.60 years, range 23–79 years) without any history of neurological or somatosensory impairment. The relevant university and hospital human ethics committees approved the study and written informed consent was obtained from each participant.

### Demographic and clinical profile

Background information included age, gender, and premorbid hand dominance (49). For the stroke patients, a clinical profile obtained within 48 h of the MRI study included the following: severity of neurological impairment, using the National Institute of Health Stroke Scale (NIHSS) (50); severity of global disability, using the Barthel Index (51); and upper limb function, using the action research arm test (ARAT) (52). Severity of somatosensory impairment was quantified across several modalities, including touch (see below); limb position sense, using the wrist position sense test (WPST) (53); tactile object recognition, using the functional tactile object recognition test (54); and temperature discrimination, using the Rolyan^®^ hot and cold discrimination kit. Age-matched healthy controls were also assessed on measures of somatosensentation.

### Quantification of touch impairment

The primary somatosensory outcome measure was the tactile discrimination test (TDT) (55), a psychophysical measure of touch discrimination of plastic gratings using the fingertip. Participants discriminate differences in finely graded plastic texture surfaces using the method of constant stimuli and a three-alternative forced-choice design. Five surface sets, which span the Weber function of texture differences, are each presented 10 times. The test score is the probability of correct discrimination response across all stimuli presented (*n* = 50) and represents the area that subtends the psychometric function after accounting for chance. The TDT has high test–retest reliability, age-appropriate normative standards, and excellent discriminative properties (55). Touch detection of the fingertips was assessed using the Weinstein enhanced sensory test (WEST) hand monofilaments and the rapid threshold procedure (56).

### Image acquisition

#### Functional Imaging Sequences

Whole-brain fMRI studies were performed using a 3-T GE Horizon LX Sigma MRI scanner with quadrature head coil (GE Medical Systems, WN, USA). Five minutes of resting-state data (100 volumes) were acquired for all participants. Images were acquired in 25 axial slices spanning cerebellum to the apex of the cerebrum using a gradient-echo, echoplanar (EPI) sequence [repetition time (TR) = 3000 ms; echo time (TE) = 40 ms; flip angle = 75°; field of view (FOV) = 240 mm; 128 × 128 matrix; slice thickness = 4 mm; interslice gap = 1 mm; in-plane voxel size = 1.95 mm × 1.95 mm; bandwidth = 100]. The participants were instructed to close their eyes and perform no particular task. Participants’ arms rested comfortably on their chest, but not touching each other or anything else. The data were collected immediately after performing an in-scanner somatosensory task involving perception of a plastic texture grating, the results of which have been reported elsewhere (3). The participants were monitored during the scanning session to ensure that they were awake and alert. They were debriefed after resting-state data collection and none of them reported falling asleep.

#### Structural Imaging Sequences

Whole-brain anatomic and angiographic images were acquired at the same session and included the following: a high-resolution 3D anatomical image, 2D T1-weighted and axial 2D T2-weighted images in the same plane as EPI, and 2D angiographic images.

### Data analysis

#### Pre-Processing of fMRI Data

Pre-processing for each participant’s data included image conversion, slice timing correction, determination of optimum realignment target (median center-of-within-brain intensity), motion detection and realignment (rigid body with six degrees of freedom), normalization to a customized EPI brain template (see below), Gaussian smoothing (8 mm full width at half maximum), and automated creation of within-brain mask of normalized images, using Statistical Parametric Mapping, SPM2 (www.fil.ion.ucl.ac.uk) and iBrain™ software (57). Motion correction parameters were included as covariates of no interest. Data from each imaging run were scaled to a grand mean of 100. The statistical analysis of the resting-state data employed an Autoregressive AR (1) model to account for temporal autocorrelation in the data.

For group analyses, fMRI data were brought into standard space. The spatial normalization target used was a custom template, approximating the EPI template in Montreal Neurological Institute (MNI) space supplied with SPM2. The custom template was created in an iterative fashion from a larger group of participants (*N* = 33) involved in the overall study. Images of patients with right hemisphere lesions were flipped such that all infarcts were in the left hemisphere.

#### Pre-Processing for Connectivity Analysis

Several processing steps were used to optimally prepare the functional data for analysis of voxel-based correlations. Data were high-pass filtered (using SPM8) (www.fil.ion.ucl.ac.uk) with a high-pass cut-off of 0.01 Hz and low-pass filtered in iBrain™ (57) using a finite impulse response filter to remove the effect of high-frequency noise (*f* < 0.08 Hz) (58).

#### Construction of Seed Regions of Interest

To measure interregional functional connectivity of the somatosensory system, we identified functionally and anatomically defined regions of interest (ROIs) representing nodes in the somatosensory system. These ROIs for functional connectivity analysis were determined by identifying regions of maximal activation from somatosensory fMRI task-related brain activation data in healthy controls (59). Significant activation clusters were restricted to the *a priori* determined cortical ROIs, the hand regions of SI, and bilateral SII, using cytoarchitectonic maps (60). The thalamic clusters were restricted to regions of the thalamus previously reported to show high probability of connectivity to somatosensory cortex, based on a thalamic connectivity atlas (61).

Six seeds were selected, and comprised clusters in the left and right primary and secondary somatosensory cortices and left and right somatosensory ventroposterior lateral thalami. Each cortical seed ROI was approximately 100 voxels in size (voxels were 1.95 mm × 1.95 mm × 4 mm in size). The cortical seed regions were constructed to make the ROIs relatively uniform in size and were anatomically verified. As the thalamic seeds were based on the thalamic atlas (61), the size was determined by that template (141 and 168 voxels). Seeds were placed on the normalized images for each individual.

#### rsfMRI Correlation Analysis

The first step in all rsfMRI analyses was to extract BOLD signal time courses from each of the six ROIs by averaging timecourses over voxels within each region for each individual at each time point. For each individual, to compute functional connectivity maps corresponding to a selected seed ROI, the average BOLD signal timecourse of the voxels within the ROI was correlated against all other voxels within the brain, as originally described by Biswal et al. (62). Several potential sources of spurious variance along with their temporal derivatives were included in the design matrix as confounds: (1) six parameters obtained by rigid body correction of head motion; (2) the average whole-brain signal; (3) signal from a ventricular cerebrospinal fluid (CSF) ROI; and (4) signal from a region centered in the white matter (63). Regions in the CSF and white matter were identified manually using MRIcro software (64). The regression of these factors as variables of no interest was aimed at removing fluctuations unlikely to be involved in specific regional correlations (63). The analysis was performed using Statistical Parametric Mapping, SPM8 (www.fil.ion.ucl.ac.uk), with the individual functional connectivity maps thresholded at *p*-value <0.001 (uncorrected) at the voxel level.

#### Second Level Imaging Analysis

In the group analysis, the contrast (con*.img) images from the individual analyses of each individual participant were combined in a second level, random-effects model. To test for differences in patterns of functional connectivity between the healthy and stroke groups, *between-group* differences were evaluated using two-sample *t* tests. To test for differences in patterns of functional connectivity within the stroke group between the 1-month and 6-month time points, *within-group* differences were evaluated using paired *t* tests. In order to identify how differences in functional connectivity over time might be associated with changes on clinical test scores, individual changes in TDT scores over time were included as a regressor in subsequent correlation analyses in the group-level random-effects analysis of change in functional connectivity for the stroke group. Only clusters with *p*-values <0.05 (false discovery rate, FDR, corrected) are reported as significant. Anatomical localization of significant clusters was defined using the anatomy toolbox in SPM8, which is based on probabilistic cytoarchitectonic maps (60).

Lesion locations were outlined on axial slices of the 3D anatomical images obtained at 6 months post-stroke, plotted into stereotactic space, as described previously (65), and displayed on a template. The percentage overlap between lesion location and the seed regions was defined for each participant.

## Results

### Demographic, lesion, and clinical data

**Figure 1:**
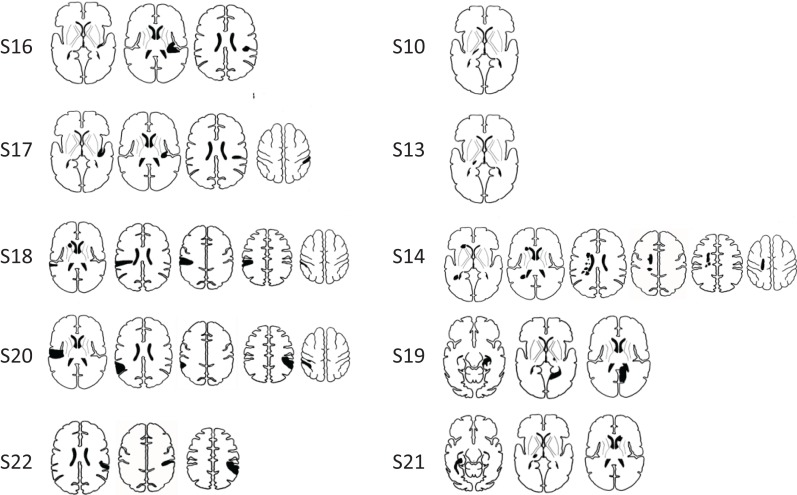
**Infarct locations for individual stroke participants**. Lesions were predominantly located in cortical somatosensory regions (SI and/or SII) (images in left column), and in somatosensory areas of the thalamus (images in right column). Infarct locations for each individual are plotted in stereotactic space. Images are displayed in neurological convention (subject’s left is displayed on image left).

Ten stroke survivors (4 male, mean age 58.96 years, range 18–79 years) were studied at approximately 1 month (*M* = 4.56, SD = 1.58 weeks) and 6 months (*M* = 26.99, SD = 1.69 weeks) post-stroke (Table 1). All were right-hand dominant with a median hand laterality quotient of 100 (49). The left hemisphere was infarcted in six patients (Figure 1). Five patients had lesions primarily involving subcortical somatosensory structures, in particular the thalamus, and five had lesions predominantly involving cortical SI and/or SII. The percentage overlap between lesion location and our pre-defined seed regions is provided in Table 1. All with subcortical lesions had involvement of thalamus, often including ventral posterolateral nucleus, a region known to project somatosensory information to SI. Only three had 2–10% overlap with the thalamic seed used in analysis. Those with cortical lesions primarily had involvement of postcentral gyrus (*n* = 3) and/or secondary somatosensory regions (*n* = 5) including parietal operculum and nearby regions of the insula and supramarginal gyrus. Across patients there was no overlap between lesion site and the SI seed. Four patients had lesion locations that overlapped with the SII seed; three had 10–19% overlap and a further patient with a very large lesion had 87% overlap.

**Table 1:** **Background and clinical characteristics and lesion details of stroke patients (*N* = 10)**.

ID	Age	Gender	Side of lesion	Site of lesion	Lesion volume (voxels)	Overlap with seed regions (%)	Weeks since stroke		NIHSS	
							1 month	6 months	1 month	6 months
S10	71	M	L	Lateral thalamus (vpl, vpm)	345	0	7.57	25.29	1	0
S13	71	F	L	Lateral thalamus (vpl, vpm)	253	5 – L Th	3.29	28.57	2	1
S14	56	M	L	Multiple lesions in hemispheric white matter	22,761	2 – L Th	6.14	25.86	6	6
S16	76	M	R	Posterior insula, inferior parietal lobule, adjacent hemispheric white matter	14,728	19 – R SII	6.00	30.86	1	1
S17	40	F	R	Posterior insula, inferior parietal lobule, postcentral gyrus	3998	19 – R SII	3.71	26.86	3	1
S18	79	M	L	Putamen/caudate nucleus, parietal/cortical	21,939	87 – L SII	3.86	26.00	4	1
S19	18	F	R	Thalamus (lp), hippocampus, fusiform gyrus	12,465	0	2.43	25.29	4	3
S20	55	F	L	Supramarginal gyrus, parietal operculum, superior parietal lobule, postcentral gyrus	6593	0	3.57	27.14	4	1
S21	63	F	L	Thalamus (vpl), occipital periventricular white matter, lacunar lesion in head of right caudate nucleus	10,107	10 – L Th	5.00	27.29	3	2
S22	59	F	R	Postcentral gyrus, superior parietal lobule, anterior portion	8990	10 – R SII	4.00	26.71	2	2
Median	59.00	4M; 6F	6L; 4R		8990		3.93	26.77	3.00	1.00
(IQR) 25th–75th	(55.00–71.00)				(2435–13,597)		(3.61–5.75)	(25.89–27.25)	(2.00–4.00)	(1.00–2.00)

ID, stroke identification number; NIHSS, National Institute of Health Stroke Scale, 1–4 = minor stroke, 5–15 = moderate stroke, 16–20 = moderate/severe stroke, and 21–42 = severe stroke (50); M, male; F, female; R, right; L, left; voxel size = 1.95 mm × 1.95 mm × 4mm; vpl, ventral posterolateral nucleus; vpm, ventral posteromedial nucleus; lp, lateral posterior nucleus; Th, thalamus; SII, secondary somatosensory cortex; IQR, interquartile range.

Patients presented with wide variation in severity of touch discrimination (Table 2), ranging from −11.58 (very severe impairment) to 79.31 (just within the normal range) on the TDT (55) at the 1-month study. Several patients performed within normal limits on the TDT at 6 months post-stroke, and in three cases at the 1-month time point. Somatosensory impairment was indicated in these patients on the basis that the TDT score for the affected hand was lower than for the “unaffected” hand, they demonstrated impairment on other clinical somatosensory tests, and/or they reported a “hyper-sensitivity” profile of *heightened* sensitivity to somatosensory stimuli.

**Table 2:** **Scores on somatosensory and hand function tests in the stroke group (*N* = 10)**.

ID	TDT – Aff (/100)		TDT – Unaff (/100)		WEST – Aff		WEST – Unaff		fTORT – Aff (/42)		Temp Aff (/10)		WPST Aff (error)		ARAT Aff (/57)	
	Initial	6 months	Initial	6 months	Initial	6 months	Initial	6 months	Initial	6 months	Initial	6 months	Initial	6 months	Initial	6 months
S10	43.10	77.59	75.37	85.22	1.1	0.2	0.135	0.035	41	40	5	9	15.40	10.90	57.0	56.0
S13	72.91	72.66	81.03	72.41	1.1	0.135	0.035	0.07	41	40	10	10	7.85	4.45	55.0	55.5
S14	30.54	47.29	67.98	81.03	102	0.07	0.07	0.07	29	40	7	9	21.75	20.30	0.0	0.0
S16	75.12	76.85	86.45	93.60	0.135	0.135	0.14	0.135	38	42	4	4	14.20	7.10	56.5	57.0
S17	8.87	40.39	52.71	77.59	300	1.1	0.035	0.035	34	42	0	2	16.65	13.90	42.0	53.5
S18	40.39	67.73	83.99	89.41	200	1.1	0.035	0.07	40	41	8	8	15.20	13.85	54.5	57.0
S19	79.31	66.99	84.73	73.64	0.035	0.035	0.07	0.035	40	42	10	10	11.15	5.35	57.0	57.0
S20	−11.58	78.57	87.93	81.53	102	0.2	0.07	0.135	40	41	0	0	17.70	12.30	49.0	57.0
S21	29.56	33.74	68.72	85.47	2	0.07	0.035	0.035	37	41	8	7	16.25	12.50	55.0	56.5
S22	−8.37	32.02	63.55	57.88	2	1.1	0.07	0.035	31	39	8	4	15.60	15.70	11.0	42.0
Median	35.47	67.36	78.20	81.28	2.00	0.17	0.07	0.05	39.0	41.0	7.5	7.5	15.50	12.40	54.75	56.25
(IQR) 25th–75th	(14.04–65.46)	(42.12–75.80)	(68.17–84.55)	(74.63–85.41)	(1.10–102.00)	(0.09–0.88)	(0.035–0.07)	(0.04–0.07)	(34.8–40.0)	(40.0–41.8)	(4.3–8.0)	(4.0–9.0)	(14.45–16.55)	(8.05–13.89)	(43.75–56.13)	(54.00–57.00)

TDT, tactile discrimination test (55), score is percentage correct area under the curve (2). The 95th percentile criterion for abnormality is a score <66.1. Minus values indicate scores with touch discrimination less than chance; WEST, Weinstein enhanced sensory test monofilaments touch detection score for the fingertip used in the TDT, threshold in grams pressure (56); fTORT, functional tactile object recognition test, score out of maximum 42, 5th percentile criterion of abnormality is 37 (54); Temp., Hot/cold temperature discrimination, number correct out of 10; WPST (average error), wrist position sense test, average error in degrees, 95th percentile criterion of abnormality is 9.5° average error (53); ARAT, action research arm test, score out of maximum 57 (52); IQR, interquartile range.

For the stroke group, mean affected-hand score on the TDT at the 1-month time point was 35.98 ± 33.13 SD (median 35.47 percentage correct area under the curve), compared to 79.85 ± 8.11 SD (median 77.09) for healthy controls in the matched hand. TDT scores were significantly higher in the healthy control group than in the patient group (Mann–Whitney *U* = 11.00, *p* = 0.002). The stroke group demonstrated significant improvement in TDT scores with the affected hand between the 1- and 6-month time points (*Z* = −2.293, *p* = 0.022). Clinical scores and demographic and clinical information for the stroke patients are presented in Table 1.

### Functional connectivity during the resting state

#### Functional Connectivity of Stroke Patients Compared to Healthy Controls

**Figure 2:**
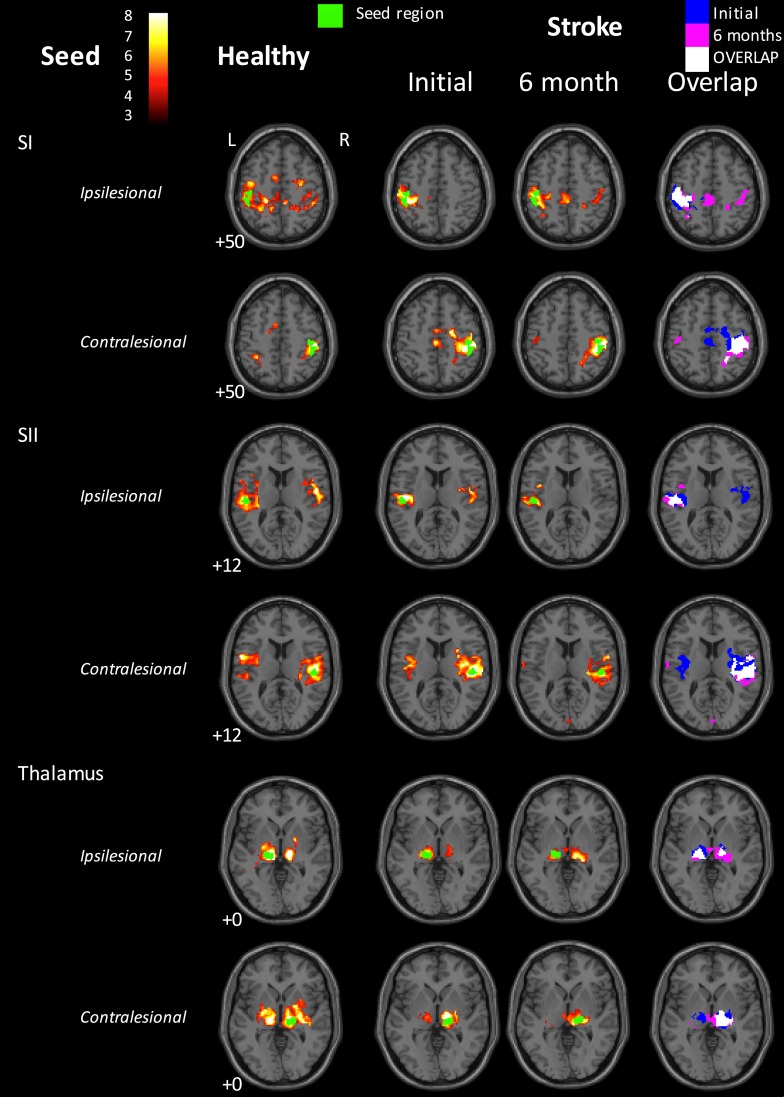
**Functional connectivity maps for the healthy control group and for the stroke group at the 1- and 6-month time points**. Left three columns: group-level functional connectivity maps. Right-hand column: overlaps of binarized group-level functional connectivity maps for the stroke group at the two time points. Blue = 1-month time point; Red = 6-month time point; Yellow = overlap. The stroke group appears to show disrupted interhemispheric functional connectivity for the SI seeds at 1 month post-stroke, relative to healthy controls. Some return of interhemispheric functional connectivity can be seen at 6 months. In contrast, interhemispheric SII connectivity in the stroke group appeared greater at 1 month than at 6 months post-stroke. The seed region is indicated in green. Images are displayed in neurological convention (subject’s left is displayed on image left). The left hemisphere represents the ipsilesional hemisphere – images of patients with right hemisphere lesions were flipped such that all infarcts are represented in the left hemisphere. Healthy controls were individually matched and images flipped accordingly. Slice numbers represent axial slice position in Montreal Neurological Institute (MNI) space. Color scale represents *Z*-values of group functional connectivity maps. SI, primary somatosensory cortex; SII, secondary somatosensory cortex. Analyses are based on contrast maps with an individual voxel height threshold level of *p* < 0.001. Results are displayed for significant clusters with *p* < 0.05 (false discovery rate, FDR, corrected).

Within the healthy control group, the SI seeds for both hemispheres showed significant functional connectivity with bilateral SI and motor (Brodmann Area, BA 4a, 6) regions (Figure 2). In contrast, at 1 month post-stroke the stroke group exhibited a lack of interhemispheric connectivity for both of the SI seeds, with each SI seed functionally connected only with surrounding SI and motor areas. At 6 months post-stroke, there appeared to be some return of interhemispheric SI connectivity for the stroke group (Figure 2). For example, the ipsilesional SI seed showed significant functional connectivity not only with surrounding SI and motor areas but also with contralesional SI, contralesional visual and motor areas, and with ipsilesional SII. Similarly, the contralesional SI seed remained functionally connected with surrounding SI and motor regions, and showed connections not present at the 1-month time point with ipsilesional SI and SII, and contralesional middle occipital gyrus. At the 1-month time point, the healthy group exhibited significantly greater functional connectivity than the stroke group between the contralesional SI seed and a cluster in the contralesional occipital lobe and contralesional cerebellum (MNI = 30/−56/16; *k* = 99 voxels; *z* = 4.76).

In the healthy control group, SII seeds of each hemisphere exhibited significant functional connectivity with bilateral SII and SI, as well as with medial supplementary motor area (SMA, BA 6). The stroke group demonstrated a similar pattern of connectivity for the ipsilesional SII seed at 1 month post-stroke. For the contralesional SII seed, significantly connected clusters also extended into bilateral SI. At 6 months post-stroke, the ipsilesional SII seed showed functional connectivity only with surrounding SII and SI. The contralesional SII seed was again functionally connected with bilateral SII and contralesional SI, with additional small clusters in contralesional SMA (BA 6) and medial visual areas (BA 17, 18, commonly referred to as human V4 and V2).

In the healthy control group, thalamus seeds of each hemisphere were functionally connected to a statistically significant extent with bilateral thalami (thalamus surrounding the seed region in the same hemisphere, as well as contralateral thalamus) and SII/insula in the same hemisphere (Figure 2). At 1 month post-stroke, the patient group showed significant functional connectivity from both thalamus seeds with bilateral thalami, although to a less extent than that seen in the healthy control group. In addition, the contralesional thalamus was functionally connected with small clusters in contralesional inferior and superior frontal gyri, and contralesional cerebellum. At 6 months, ipsilesional thalamus in stroke patients still showed significant functional connectivity with thalami in both hemispheres, whereas the contralesional thalamus was only functionally connected with surrounding contralesional thalamus and with a small cluster in the left putamen.

#### Longitudinal Functional Connectivity Changes in the Stroke Group

To test for differences in patterns of functional connectivity within the stroke group between the 1- and 6-month time points, within-group differences were evaluated using paired *t* tests (Table 3). For the contralesional SI seed, there was significantly greater functional connectivity at 1-month than at 6-month post-stroke between contralesional SI and a cluster falling in the contralesional cerebellum and hippocampus. At 6 months, there was significantly greater functional connectivity between ­contralesional thalamus and a cluster in ipsilesional middle cingulate cortex.

**Table 3:** **Functional connectivity changes in the stroke group between the 1- and 6-month time points, and changes associated with improvement in TDT scores**.

Seed region	Cluster size (voxels)	*Z*-value	MNI maxima coordinates (*x, y, z*)	Cluster anatomical location of significantly correlated regions
**Regions showing greater functional connectivity at 1 month**				
Contralesional SI	60	4.14	14, −32, −18	Contralesional cerebellum lobules I–V, hippocampus
			12, −28, −6	
			6, −34, −20	
**Regions showing greater functional connectivity at 6 months**				
Contralesional thalamus	49	4.44	−16, −22, 38	Ipsilesional middle cingulate
**Regions showing greater functional connectivity at 6 months than 1 month post-stroke in association with improvement in touch discrimination**				
Contralesional SII	53	4.55	52, −58, 26	Contralesional IPC
	30	5.02	58, −26, −10	Contralesional middle temporal gyrus
Contralesional thalamus	35	4.27	12, −42, −40	Contralesional cerebellum lobule IX
			20, −44, −34	
**Regions showing greater functional connectivity at 1 month than 6 months post-stroke in association with improvement in touch discrimination**				
Contralesional SI	42	3.89	28, −44, −24	Contralesional cerebellum lobules V, VI

Anatomical definitions are based on the anatomy toolbox in SPM8, which is based on probabilistic cytoarchitectonic maps (60).

MNI, Montreal Neurological Institute; SI, primary somatosensory cortex; SII, secondary somatosensory cortex; IPC, inferior parietal cortex.

#### Functional Connectivity Changes Associated with Somatosensory Improvement

**Figure 3:**
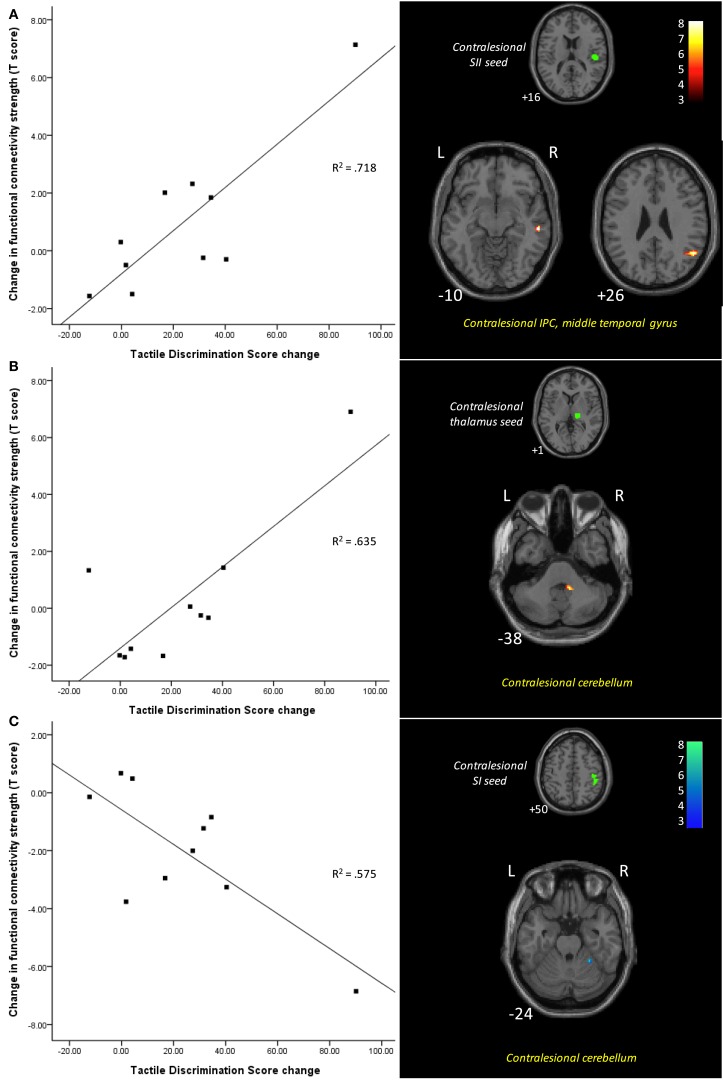
**Functional connectivity differences between 1 and 6 months post-stroke associated with changes in tactile discrimination test (TDT) scores**. Scatter plots illustrate change in TDT score plotted against the difference between the two time points in connectivity strength between the seed and the cluster (shown in images on the right), for each individual. Improvement in TDT scores was associated with **(A)** greater functional connectivity at 6 months between the contralesional SII seed and clusters in the contralesional inferior parietal cortex and contralesional middle temporal gyrus; **(B)** greater functional connectivity at 6 months between the contralesional thalamus seed and a cluster in contralesional cerebellum; and **(C)** less functional connectivity at 6 months between the contralesional SI seed and contralesional cerebellum. Images are displayed in neurological convention (subject’s left is displayed on image left). The left hemisphere represents the ipsilesional hemisphere – images of patients with right hemisphere lesions were flipped such that all infarcts are represented in the left hemisphere. Slice numbers represent axial slice position in Montreal Neurological Institute (MNI) space. Color scale represents *Z*-values of functionally connected clusters associated with TDT score change. SI, primary somatosensory cortex; SII, secondary somatosensory cortex; IPC, inferior parietal cortex. Analyses are based on contrast maps with an individual voxel height threshold level of *p* < 0.001. Only clusters with *p*-values <0.05 (false discovery rate, FDR, corrected) are reported as significant and displayed.

In subsequent correlation analyses, changes over time in clinical scores, as measured using the TDT, were included as a regressor in the group-level random-effects analysis of change in functional connectivity for the stroke group. The functional connectivity changes significantly associated with changes in TDT scores are shown in Table 3 and Figure 3. Greater improvement in TDT scores was associated with greater functional connectivity at 6-month than at 1-month post-stroke between the contralesional SII seed and clusters in the contralesional IPC and contralesional middle temporal gyrus. Greater connectivity at 6-month than at 1-month post-stroke between the contralesional thalamus seed and a cluster in contralesional cerebellum was also associated with greater improvement. Greater functional connectivity at 1-month than at 6-month post-stroke between the contralesional SI seed and contralesional cerebellum was associated with greater improvement in TDT scores over time. Conversely, relative to the 6-month recovery time, greater improvement in TDT scores may be viewed as being associated with less functional connectivity at 6 months than at 1 month between the contralesional SI seed and contralesional cerebellum (Figure 3).

## Discussion

### Interhemispheric functional connectivity is disrupted at 1 month after stroke and shows some recovery toward normal levels at 6 months

Our findings of functional connectivity extend previous findings of changes in activation of brain regions with somatosensory impairment and add to the growing body of literature on the role of interhemispheric connectivity in stroke recovery across a range of functions. One month post-stroke, patients with impaired touch sensation and lesions predominantly located in somatosensory areas of the thalamus, and/or in cortical somatosensory regions (SI and/or SII), exhibited disruption of interhemispheric functional connectivity of homologous SI regions relative to age-matched healthy controls. At 1 month post-stroke, the stroke group only exhibited SI functional connectivity with SI within the same hemisphere; at 6 months, there was some return of interhemispheric SI connectivity.

Our finding of less interhemispheric connectivity in the stroke patients with impaired touch sensation relative to healthy controls early post-stroke is consistent with evidence of disrupted interhemispheric functional connectivity in stroke patients for other functions, such as movement and attention (7, 25, 27, 28). The disruption observed is likely to be behaviorally relevant, given that activity in both hemispheres has been shown to be important in sensory processing (31, 66) and in activation studies of somatosensory and motor recovery (33–36). Further, previous studies using rsfMRI in stroke recovery across functions have highlighted the importance of interhemispheric functional connectivity in behavioral performance and in recovery over time (7, 25, 28).

Evidence of SI interhemispheric connectivity at 6 months in stroke patients with less severe touch impairment is consistent with growing evidence from related studies. Activation studies of motor recovery indicate “return to more normal patterns” is associated with better recovery in the post-acute and chronic phase (e.g., 6 months) post-stroke (67, 68). In addition, a recent review of rsfMRI studies in motor recovery found that reorganization of motor networks encompasses a restoration of interhemispheric functional coherence in the resting state, particularly between the primary motor cortices (27). While we do not report a significant longitudinal change in connectivity between contralesional and ipsilesional SI, we did observe significant interhemispheric connectivity for both SI seeds at 6 months that was not present at 1 month. Together, our findings resonate with studies illustrating the role of inhibitory influences from intact hemisphere in stroke recovery (33) and highlight the need to re-establish a balance of activity across hemispheres in association with ­improvement (35).

### Disruption and resolution of functional connectivity with occipital visual areas

Another key finding was the role of functional connectivity with primary visual occipital regions. At the 1-month time point, functional connectivity between contralesional SI and the occipital lobe was significantly less in the stroke group compared to the matched healthy control group. At the 6-month time point, the stroke group demonstrated functional connections with visual occipital areas that were not present at 1 month post-stroke, including between ipsilesional SI and contralesional visual areas (BA 17, 18), between contralesional SI and contralesional middle occipital gyrus, and between contralesional SII and bilateral visual areas (BA 17, 18). Together, these findings suggest a pattern of disruption of functional connections between somatosensory and visual areas at 1 month post-stroke, which showed some return after 6 months.

Supporting the suggestion of less connectivity with visual occipital regions early post-stroke is Park et al.’s (30) finding that one month after stroke, patients with motor impairment demonstrated decreased functional connectivity between primary motor regions and occipital cortex. Similarly, Carey et al. (3) reported that in a group of stroke patients with thalamic lesions studied at 1 month post-stroke, touch discrimination correlated negatively with task-related activation in occipital regions. Connectivity with occipital regions at 6-month are also consistent with Seitz et al.’s (41) study of the functional networks related to motor recovery, which found that improved motor function after stroke was associated with involvement of distributed areas including extrastriate visual areas. Thus, there seems to be a pattern of disrupted interactions between ­sensorimotor and visual occipital systems around 1 month after stroke, with some resolution over time that may be clinically relevant.

### Functional connections to frontoparietal attention regions

In stroke patients, functional connections to frontoparietal attention regions (69), involving middle cingulate and IPC, were significantly greater at 6 months than at 1 month post-stroke, and these differences between time points were in part associated with changes in behavioral performance. Functional connectivity between contralesional thalamus and ipsilesional middle cingulate cortex was significantly greater at 6 months than at 1 month post-stroke. Furthermore, behavioral improvement on the TDT was associated with greater functional connectivity 6 months post-stroke between contralesional SII and a cluster in contralesional IPC. In addition, the individuals who showed thalamocortical functional connectivity with frontal regions at the 1-month time point also had relatively low TDT scores, while those who showed this connectivity pattern at 6 months had better TDT scores.

Activation of distributed attention networks has been observed in previous task-based studies of stroke recovery, including in relation to somatosensory recovery (3, 36). Involvement of frontoparietal attention networks in association with behavioral outcome has been a common finding in stroke patients in the motor domain (46–48). Further, longitudinal changes in rsfMRI include changes in frontal and parietal cortices during motor recovery (30). Here, we extend this finding of functional connectivity to somatosensory recovery post-stroke. Baseline brain activity in the medial thalamus and the frontoparietal network is important in perception (70) and may affect information processing following sensory impairment. In addition, focal attention involved in perception of pain, processing of reward, and error detection, has been associated with activity in medial frontal/anterior cingulate (69). Attention is essential to any perception or learning (69, 71), and has been identified as a key element of recovery from brain injury (25, 42, 45). It could be speculated that our findings reflect stroke patients’ use of higher-level attention and behavioral processes to supplement previously more automatic somatosensory perceptual functions.

### Involvement of contralesional hemisphere

Changes in functional connectivity between the 1- and 6-month time points and in association with improvement in TDT over time were all seeded within the contralesional hemisphere, i.e., contralesional SI at 1 month and contralesional thalamus and SII at 6 months. We did not find significant changes over time in connectivity from our ipsilesional seeds. Further, the regions showing relatively increased correlation were also primarily in the contralesional hemisphere, with the exception of ipsilesional middle gyrus at 6-month >1-month post-stroke. These findings highlight a role for change in connectivity of the “intact” contralesional hemisphere, in particular somatosensory SI, SII and thalamus regions, in individuals with impaired touch sensation post-stroke. Further, the observation that increased connectivity from these contralesional somatosensory seeds was associated with improvement in touch discrimination scores over time suggests a role for the contralesional somatosensory network in facilitating touch discrimination perception. While previous task-based fMRI studies typically show an initial increase in activation of contralesional sensorimotor cortex early followed by restoration of activation in the ipsilesional cortex, our finding suggests that disruption of the initial interhemispheric connectivity at resting state may lead to ongoing alterations in the activity (functional connectivity) of contralesional hemisphere. These relative increases in connectivity, observed both early and late, may help in achieving a more balanced interhemispheric connectivity in association with greater improvement in patients with partial recovery.

At 1-month, increased connectivity between contralesional SI and contralesional cerebellum was associated with greater improvement in touch sensation over time. In comparison, at 6 months, the relatively greater connectivity associated with better touch discrimination was between contralesional SII and IPC and contralesional thalamus and cerebellum. Interestingly, contralesional cerebellum had changed connectivity to somatosensory seeds associated with improvement at both times, but via different nodes of the network. A role for increased connectivity between contralesional SI and cerebellum, at 1-month associated with improvement, is consistent with our observation of greater connectivity between these regions in the healthy group, compared to stroke patients, at 1-month. Longitudinal changes in rsfMRI during motor recovery have also involved bilateral thalamus and cerebellum, with involvement of cerebellum persisting over the 6-month period post onset (30). A large proportion of cerebellum maps to association areas (72). In addition, the cerebellum has connections with SI, although preferentially with the contralateral cerebrum (72). Afferent projections first synapse in the deep cerebellar nuclei and then project to a second synapse in the contralateral thalamus that in turn serves as a relay to the cerebral cortex, consistent with involvement of thalamus at 6 months. Co-observation of greater functional connectivity of contralesional thalamus with ipsilesional middle cingulate at 6 months, suggests an increased interhemispheric connectivity. Involvement of contralesional thalamus has been reported in association with touch impairment in a sample of 19 stroke survivors at 1-month post-stroke (3). Contralesional thalamus has potential to be accessed irrespective of lesion location (3), has an influence on bilateral SI via its prefrontal connections (73), and may have a role in gating of sensory information and in large-scale reorganization in the somatosensory cortex and thalamus after sensory loss (74, 75).

### Limitations

The major limitation of this study was the small and heterogeneous sample of stroke patients. Replication of these preliminary findings in larger samples is required. Use of a larger sample would also allow investigation of these changes without the need to flip individual brain maps into common space. This would permit inferences about the role of lateralized frontoparietal attention networks in facilitating post-stroke behavioral improvement (8). While it is recognized that functional connectivity may be influenced by the participants recent experience (76), the sequence of acquisition was common for all participants, i.e., it was immediately preceded by a touch discrimination task. Further, our stroke findings may be interpreted with reference to healthy controls who underwent the same protocol sequence, and our longitudinal findings with reference to connectivity studies in the same individual over time.

Application of rsfMRI analyses in stroke patients presents issues that need to be considered in the interpretation of our findings. The potential impact of lesion location on pre-defined seed ROIs is an unavoidable issue. This was in part minimized through application of individual lesion masks during the normalization phase. In addition, we quantified the percentage overlap between the lesion and seed region for each participant to monitor the presence of this potential limitation. All but one participant had <20% overlap. There was no overlap with the ipsilesional SI seed and only 10% or less overlap with the thalamic seed. The seed with most overlap was the SII seed, with 4 of 10 patients having overlap. Our major findings of change in connectivity were evident for contralesional seeds, and thus, can be interpreted with confidence. Further, lack of evidence of significant change for ipsilesional SI and thalamic seeds is unlikely explained by analysis method and seed overlap, as this was minimal. Interpretation of functional connectivity from ipsilesional SII may be impacted by overlap between lesion and seed. Although we did not find a significant change over time, we did observe significant connectivity from ipsilesional SII at 1 and 6 months, suggesting presence of lesion overlap with this seed is an unlikely explanation. A recent investigation of overlap between lesion location and seeds between stroke and healthy groups suggests that the percent of infarct-related overlap to any ROI was not related to connectivity strength in connections that included those damaged seeds (77). While this finding is based on a larger sample (*n* = 32) and multiple correlations, it does provide some support for interpretation of seed-based connectivity data in stroke patients. Finally, even if the differences in connectivity observed between stroke and healthy controls is due to impaired anatomic connections from these regions, our findings still inform us of the key functional connections involved in somatosensory impairment, the impact of lesion on the function, and the changes in functional connectivity associated with clinical improvement in touch discrimination.

Use of the BOLD signal in fMRI studies of stroke patients has been a highly debated issue given the potential impact of vascular compromise. The BOLD signal provides an indirect indication of neural activity, and changes in resting-state activity can reflect a complex combination of neural, vascular, and metabolic factors (78). Connectivity analysis methods have the advantage that they do not rely on BOLD signal stability, nor assume a common hemodynamic response function (79). However, they are not immune to issues associated with abnormal neurovascular coupling in stroke patients. Indeed, it is unclear how potential vascular latency differences between brain regions impact interpretation following stroke. For example, changes in peri-infarct regions, such as hypoperfusion and potential decoupling of the neurovascular response (80), may impact the signal. It has been suggested that differences across regions may confound studies of whole-brain connectivity (81). A few studies have therefore adjusted for non-neural vascular latency differences prior to resting-state connectivity analyses in healthy controls with only a minor impact on their findings (81). However, we should exercise caution when interpreting findings in stroke patients, particularly in locations close to the lesion border. Further, it is important to recognize that changes observed with rsfMRI may reflect an interaction between neural activity and vascular changes over 1–6 months. It should also be noted that we did not exclude patients with conditions that may impact the BOLD signal, such as leukoencephalopathy and/or carotid artery disease, and thus the impact of these conditions if present is unknown.

### Implications and future directions

In summary, stroke patients showed changes in functional connectivity over a period of recovery under non-specific rehabilitation conditions. Further, most changes in functional connections from 1 to 6 months post-stroke were shown to relate to improvement in touch discrimination scores over time, in patients with partial recovery. There appeared to be some return of functional connections over time in patients between homologous SI regions, and between somatosensory and visual occipital areas, although not to the levels seen in age-matched controls. Change in connectivity over time and/or in association with improvement was observed in relation to contralesional somatosensory seeds, and primarily involved frontoparietal attention regions and cerebellum. Change in contralesional SI connectivity was important at 1-month in relation to improvement over time, while changes in connectivity of contralesional SII and thalamus become important at 6 months.

These changes in connectivity could represent future targets for therapy. In particular, increase in strength in connections between somatosensory regions and attention and vision regions is consistent with pre-existing connections with these networks and suggest targets for neuroscience-based rehabilitation approaches designed to access viable brain networks (36). While our findings indicate that some individuals spontaneously access these regions in association with improved performance, the potential exists for knowledge of these individual differences to guide access in other stroke survivors through therapy. For example, the effective sensory discrimination training approach described by us to achieve stimulus specific improvements in touch discrimination (82) employs training strategies to achieve cross-modal calibration of perceived texture roughness across touch and vision, as well as use of attentive exploration of textured stimuli and deliberate use of anticipation trials (36, 82). These strategies may be helpful in accessing vision and attention networks in survivors who may not otherwise make these connections.

Targeting of contralesional and distributed networks via secondary somatosensory cortex and thalamus is also suggested. Our findings first highlight the role of the contralesional hemisphere in post-stroke performance and recovery. The seed-based change in contralesional functional connectivity is consistent with structural and functional connectivity studies of sensorimotor training that suggest global network efficiency is influenced by long-range connections across hemispheres, in addition to ipsilesional integrity (83). Changes in connectivity of contralesional SII and thalamus at 6 months suggest a role for nodes that have connections within the somatosensory network and beyond. SII has strong connections with SI, thalamus, and homologous SII, as well as with frontal and parietal networks (84). SII has more dense bilateral connectivity than SI (85), is involved in tactile working memory, discrimination, and perceptual learning (86–88), and is regarded as an integration node of the somatosensory network. Enhanced SII connections with IPC and middle temporal gyrus at 6 months highlight connectivity with distributed networks. The potential exists to influence this highly connected node of the somatosensory network through rehabilitation designed to access discriminative and tactile learning functions. The thalamus is also implicated. It has an important role in gating somatosensory input and deactivation of contralesional thalamus is associated with touch discrimination performance in stroke survivors at 1 month post-stroke (3). Involvement of thalamus is consistent with evidence from animal studies (74, 75) that gating of sensory inputs, rather than cortical representation alone, is important in recovery. In addition, increased connectivity between thalamus and cerebellum suggests short-range functional connectivity of subcortical networks (89). Thalamus and cerebellum are two of three major subcortical network hubs identified (89). Involvement of both long-range and short-range functional connectivity changes may reflect not only the individual variation in recovery and underlying mechanisms but also the potential to drive one or other through appropriately targeted therapy. While connectivity-based research is still in its infancy post-stroke, it has great potential to guide the development of scientifically informed rehabilitation interventions.

## Conflict of Interest Statement

The authors declare that the research was conducted in the absence of any commercial or financial relationships that could be construed as a potential conflict of interest.
